# Person Perception from Face and Voice

**DOI:** 10.5334/pb.ao

**Published:** 2014-05-20

**Authors:** Serge Brédart

**Affiliations:** 1 Department of Psychology, Cognition and Behaviour (B-32), University of Liège, Belgium

In the 1980s several information processing models of face recognition were designed by cognitive psychologists. Among them, the famous Bruce and Young (1986) model appeared as particularly seminal. Following the publication of that model, a huge number of studies were conducted by psychologists and neuropsychologists in order to test the model with both healthy people and brain-injured patients. Although several amendments to the original model were proposed during the last 25 years, the general architecture of the Bruce and Young model is still considered as a useful theoretical framework guiding empirical research (see Hanley, 2011).

The interest for voice recognition, or person recognition from the voice, started a little later. These first models proposed in the 1990s (e.g., Ellis, Jones, & Mosdell, 1997) were widely and explicitly inspired by the Bruce and Young model. Most of these models applied to voice recognition the processing stages that were earlier considered for face recognition (i.e., structural encoding / recognition / access to semantic information / access to name). However, despite some pioneering works published in the 1990s, it is rather recently that face and voice recognition were narrowly compared. Making such a comparison in the first paper of the present special issue, Catherine Barsics shows that the retrieval of both semantic and episodic information is usually easier from faces than from voices. Evelyne Moyse compares age estimation from faces and voices and shows that there are similarities and differences between both. An own-age bias may exist for age estimation from both faces and voices, but age is less precisely estimated from voices than from faces.

In addition to a comparison of the specific properties of face and voice processing, the necessity to understand how voice and face information are integrated during person recognition or emotional processing became progressively obvious, and drew the attention of cognitive psychologists, neuropsychologists and cognitive neuroscientists. Sarah Stevenage and Greg Neil examine how face processing and voice processing interact during person recognition, notably through the analysis of interference between the face and voice pathways. Pierre Maurage and Salvatore Campanella analyse the alteration of face-voice integration during the processing of emotional stimuli in alcohol-dependent people. Finally, Guido Gainotti considers the patterns of disorders of familiar people recognition in patients with anterior temporal lesions including prosopagnosia, phonagnosia and multimodal recognition disorders.

The papers presented in this special issue (excepted Evelyne Moyse’s paper) are based on oral communications presented at a symposium held at the Royal Academy on November 22, 2013. This symposium was co-organised by Salvatore Campanella (ULB), Gilles Pourtois from (Gent U) and myself.

I will end this short introduction by acknowledging our sponsors, i.e., the “Académie Royale des Sciences, des Lettres et des Beaux Arts de Belgique”, the Brussels region (Visit Brussels), the “Fonds National de la Recherche Scientifique” (FNRS-FRS), the “Fonds Wetenschappelijk Onderzoek – Vlaanderen” (FWO), the “Koninklijke Vlaamse Academie van België voor Wetenschappen en Kunsten”, the “Service des relations extérieures et communication” of the University of Liège, and the Royal Academies for Science and the Arts of Belgium.

Thank you to the members of the National Committee for Psychological Sciences for their confidence, a special thank for the Presisent, Leni Verhofstadt-Denève for her encouragement and her help. I also would like to thank the co-organizers of the symposium, Salvatore Campanella and Gilles Pourtois. Last but not least, thanks to Etienne Quertemont, the former Editor-in-Chief of *Psychologica Belgica*.
